# Supplementary figures and images for: The antagonistic mechanism of Bacillus velezensis ZW10 against rice blast disease: Evaluation of ZW10 as a potential biopesticide
Source: PLoS One. 2021 Aug 27;16(8):e0256807. doi: 10.1371/journal.pone.0256807 (PMC8396770; doi:10.1371/journal.pone.0256807)

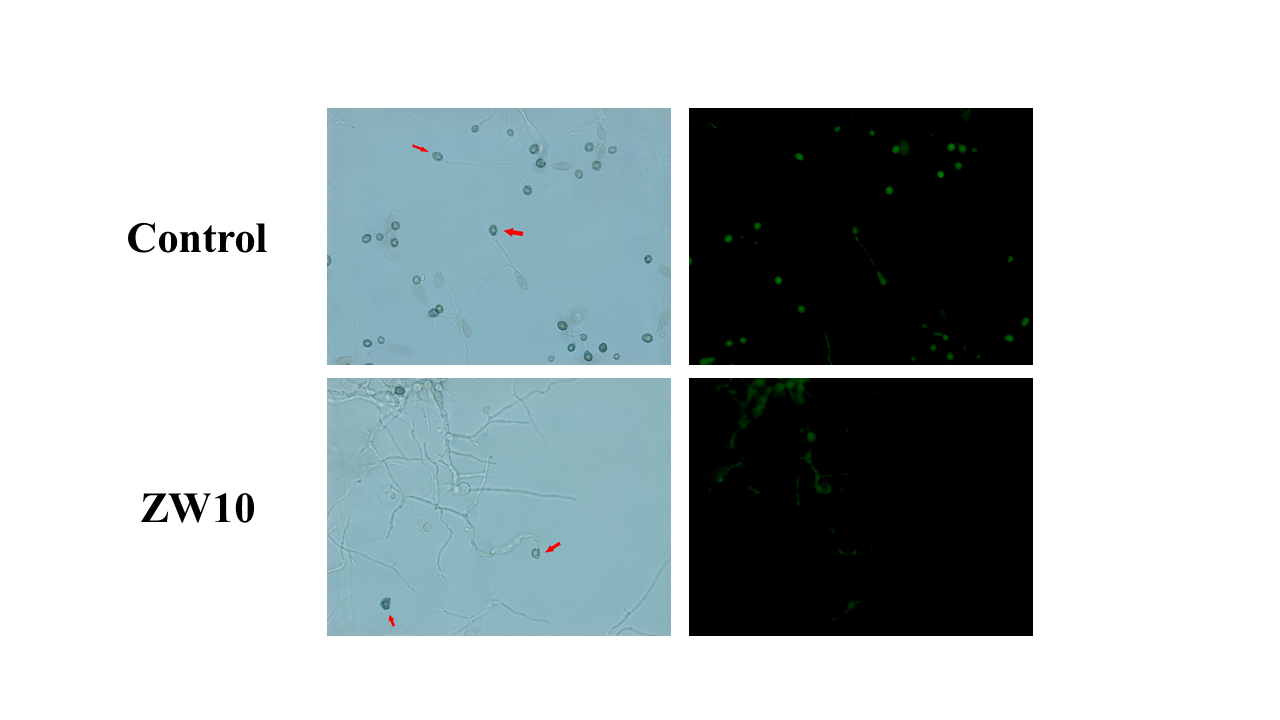

Supplement: S1 Fig — After ZW10 treatment, the appressorium was deformed and did not emit green fluoresce. (TIF) [file pone.0256807.s001.tif]
