# Supplementary material for: The antagonistic mechanism of Bacillus velezensis ZW10 against rice blast disease: Evaluation of ZW10 as a potential biopesticide
Source: PLoS One. 2021 Aug 27;16(8):e0256807. doi: 10.1371/journal.pone.0256807 (PMC8396770; doi:10.1371/journal.pone.0256807)
Supplement: S1 File — Row date for Fig 2. (PDF) [file pone.0256807.s002.pdf]

Germination rate /%					
water	Test 1	Test 2	Test 3	Average	SD
2h	86.61	88.1	91.43	88.71333333	2.014982768
8h	97.37	98.26	98.25	97.96	0.417212975
12h	98.68	96.27	95.87	96.94	1.241155376
24h	95.92	97.78	99.16	97.62	1.327554142
48h	100	96.9	95.9	97.6	1.745470328
0.1% CFB	Test 1	Test 2	Test 3	Average	SD
2h	5.1	2.02	4.5	3.873333333	1.333199993
8h	96.36	92.71	89.63	92.9	2.750793825
12h	92.06	94.01	91	92.35666667	1.246604277
24h	92.38	97.27	94.35	94.66666667	2.00885263
48h	94.12	96.75	93.26	94.71	1.484609938

Formination rate /%					
water	Test 1	Test 2	Test 3	Average	SD
2h	0	0	0	0	0
8h	85.09	86.96	84.2	85.41666667	1.150198051
12h	92.31	91.34	92.56	92.07	0.526181211
24h	94.9	94.07	97.48	95.48333333	1.451948882
48h	92.93	91.75	93.44	92.70666667	0.707782138
0.1% CFB	Test 1	Test 2	Test 3	Average	SD
2h	0	0	0	0	0
8h	5.45	3.13	4.44	4.34	0.949771902
12h	16.52	15	18	16.50666667	1.22478116
24h	17.78	18.18	20.97	18.97666667	1.418927608
48h	17.26	17.64	19.39	18.09666667	0.927589469
