# Supplementary material for: The antagonistic mechanism of Bacillus velezensis ZW10 against rice blast disease: Evaluation of ZW10 as a potential biopesticide
Source: PLoS One. 2021 Aug 27;16(8):e0256807. doi: 10.1371/journal.pone.0256807 (PMC8396770; doi:10.1371/journal.pone.0256807)
Supplement: S2 File — Row date for Fig 5. (PDF) [file pone.0256807.s003.pdf]

OsLYP6									
Wells	Reporter	ACTIN	SD Avg Ct	LYP	SD Avg Ct	Δ Ct	Rel Exp	Avg Rel Exp	SD RX
CK 0	SYBR	28.27	0.43	33.96	0.59	5.69	TRUE	1.00	0.00
		27.95		32.80		4.84	1.00		
	SYBR	27.42		33.58		6.16	1.00		
CK 24	SYBR	29.26	0.12	36.73	0.38	7.46	0.27	0.20	0.07
		29.15		37.15		8.01	0.18		
	SYBR	29.02		37.49		8.47	0.13		
CK 48	SYBR	31.32	0.19	37.10	0.22	5.78	0.86	1.16	0.30
		31.40		36.75		5.36	1.15		
	SYBR	31.68		36.69		5.01	1.46		
CK 72	SYBR	28.89	0.81	35.07	0.47	6.18	0.65	0.67	0.21
		27.48		34.13		6.65	0.47		
	SYBR	28.85		34.57		5.72	0.90		
ZW10 0	SYBR	25.73	0.34	33.73	0.51	8.00	0.18	0.16	0.04
\		25.31		34.04		8.73	0.11		
	SYBR	25.06		33.04		7.98	0.19		
ZW10 24	SYBR	25.73	0.42	32.60	0.32	6.87	0.40	0.50	0.13
		25.30		31.97		6.66	0.47		
	SYBR	26.14		32.33		6.19	0.65		
ZW10 48	SYBR	27.24	0.18	30.33	0.24	3.09	5.54	5.56	0.22
		27.07		30.10		3.03	5.79		
	SYBR	27.43		30.57		3.14	5.35		
ZW10 72	SYBR	27.42	0.25	34.60	0.53	7.18	0.33	0.24	0.07
		27.36		35.26		7.90	0.20		
	SYBR	27.82		35.65		7.84	0.21		

OsPR10a									
Wells	Reporter	ACTIN	SD Avg Ct	PR10a	SD Avg Ct	Δ Ct	Rel Exp	Avg Rel Exp	SD RX
CK 0	SYBR	28.27	0.43	30.48	0.41	2.21	TRUE	1.00	0.00
		27.95		30.44		2.49	1.00		
	SYBR	27.42		31.18		3.75	1.00		
CK 24	SYBR	29.26	0.12	31.75	0.73	2.49	8.41	13.28	5.63
		29.15		31.13		1.98	11.97		
	SYBR	29.02		30.30		1.28	19.45		

CK 48	*SYBR*	31.32	*0.19*	32.52	*0.61*	*1.20*	*20.53*	*15.08*	*4.82*
		31.40		33.22		*1.82*	*13.36*		
	SYBR	31.68		33.73		*2.06*	*11.36*		
CK 72	*SYBR*	28.89	*0.81*	33.76	*0.82*	*4.87*	*1.62*	*1.85*	*0.24*
		27.48		32.17		*4.70*	*1.82*		
	SYBR	28.85		33.34		*4.49*	*2.10*		
ZW10 0	*SYBR*	25.73	*0.34*	30.83	*0.46*	*5.11*	*1.37*	*1.42*	*0.44*
\		25.31		29.95		*4.65*	*1.89*		
	SYBR	25.06		30.61		*5.55*	*1.01*		
ZW10 24	*SYBR*	25.73	*0.42*	27.13	*0.26*	*1.40*	*17.93*	*17.17*	*2.02*
		25.30		26.97		*1.67*	*14.88*		
	SYBR	26.14		27.47		*1.34*	*18.70*		
ZW10 48	*SYBR*	27.24	*0.18*	26.23	*0.19*	*-1.01*	*95.08*	*89.58*	*4.78*
		27.07		26.20		*-0.87*	*86.38*		
	SYBR	27.43		26.54		*-0.89*	*87.28*		
ZW10 72	*SYBR*	27.42	*0.25*	30.49	*0.17*	*3.06*	*5.66*	*6.63*	*0.95*
		27.36		30.18		*2.82*	*6.69*		
	SYBR	27.82		30.46		*2.64*	*7.55*		

OsPR1a									
Wells	Reporter	**ACTIN**	SD Avg Ct	**PR10a**	SD Avg Ct	**Δ Ct**	Rel Exp	**Avg Rel Exp**	SD RX
CK 0	*SYBR*	28.27	*0.43*	38.74	*0.37*	*10.46*	*TRUE*	*1.00*	*0.00*
		27.95		39.19		*11.24*	*1.00*		
	SYBR	27.42		39.46		*12.04*	*1.00*		
CK 24	*SYBR*	29.26	*0.12*	37.17	*0.50*	*7.91*	*0.20*	*0.28*	*0.07*
		29.15		36.40		*7.25*	*0.31*		
	SYBR	29.02		36.23		*7.20*	*0.32*		
CK 48	*SYBR*	31.32	*0.19*	39.26	*0.10*	*7.94*	*0.19*	*0.20*	*0.02*
		31.40		39.38		*7.99*	*0.19*		
	SYBR	31.68		39.45		*7.78*	*0.22*		
CK 72	*SYBR*	28.89	*0.81*	35.57	*0.47*	*6.68*	*0.46*	*0.42*	*0.10*
		27.48		34.70		*7.22*	*0.32*		
	SYBR	28.85		35.43		*6.57*	*0.50*		
ZW10 0	*SYBR*	25.73	*0.34*	35.10	*0.62*	*9.37*	*0.07*	*0.07*	*0.02*
\		25.31		35.30		*9.99*	*0.05*		
	SYBR	25.06		34.13		*9.07*	*0.09*		

ZW10 24	SYBR	25.73	0.42	32.13	0.15	6.40	0.56	0.55	0.10
		25.30		32.04		6.74	0.44		
	SYBR	26.14		32.34		6.21	0.64		
ZW10 48	SYBR	27.24	0.18	32.22	0.14	4.98	1.49	1.54	0.06
		27.07		32.03		4.96	1.52		
	SYBR	27.43		32.30		4.87	1.62		
ZW10 72	SYBR	27.42	0.25	36.35	0.73	8.92	0.10	0.13	0.05
		27.36		35.36		8.00	0.18		
	SYBR	27.82		36.78		8.96	0.09		

OsPOD									
Wells	Reporter	**ACTIN**	SD Avg Ct	**POD**	SD Avg Ct	**Δ Ct**	Rel Exp	**Avg Rel Exp**	SD RX
CK 0	SYBR	28.27	0.43	28.86	0.34	0.59	TRUE	1.00	0.00
		27.95		28.25		0.30	1.00		
	SYBR	27.42		28.28		0.85	1.00		
CK 24	SYBR	29.26	0.12	28.84	0.39	-0.42	63.28	76.88	14.71
		29.15		28.48		-0.66	74.86		
	SYBR	29.02		28.05		-0.97	92.50		
CK 48	SYBR	31.32	0.19	34.22	0.38	2.90	6.34	5.28	0.94
		31.40		34.77		3.37	4.56		
	SYBR	31.68		34.93		3.26	4.95		
CK 72	SYBR	28.89	0.81	32.88	0.40	3.99	2.97	3.13	1.36
		27.48		32.14		4.67	1.86		
	SYBR	28.85		32.23		3.37	4.56		
ZW10 0	SYBR	25.73	0.34	26.69	0.33	0.97	24.18	23.81	0.62
\		25.31		26.34		1.03	23.09		
	SYBR	25.06		26.03		0.97	24.17		
ZW10 24	SYBR	25.73	0.42	27.15	0.16	1.42	17.64	17.61	3.08
		25.30		27.01		1.70	14.51		
	SYBR	26.14		27.33		1.19	20.67		
ZW10 48	SYBR	27.24	0.18	25.42	0.14	-1.82	166.29	173.86	8.36
		27.07		25.20		-1.87	172.46		
	SYBR	27.43		25.47		-1.95	182.83		
ZW10 72	SYBR	27.42	0.25	30.18	0.24	2.76	6.99	7.34	0.31
		27.36		30.00		2.64	7.58		
	SYBR	27.82		30.48		2.66	7.46		

OsPAL1										
Wells	Reporter	ACTIN	SD Avg Ct	PAL	SD Avg Ct	Δ Ct	Rel Exp	Avg Rel Exp	SD RX
CK 0	SYBR	28.27	0.43	36.88	0.36	8.60	TRUE	1.00	0.00
		27.95		36.19		8.24	1.00		
	SYBR	27.42		36.36		8.94	1.00		
CK 24	SYBR	29.26	0.12	38.84	0.38	9.58	0.06	0.08	0.01
		29.15		38.36		9.22	0.08		
	SYBR	29.02		38.08		9.06	0.09		
CK 48	SYBR	31.32	0.19	39.77	0.25	8.45	0.13	0.13	0.01
		31.40		40.02		8.63	0.12		
	SYBR	31.68		40.27		8.59	0.12		
CK 72	SYBR	28.89	0.81	35.76	0.63	6.88	0.40	0.40	0.06
		27.48		34.58		7.11	0.34		
	SYBR	28.85		35.53		6.68	0.46		
ZW10 0	SYBR	25.73	0.34	34.30	0.22	8.58	0.12	0.11	0.01
\		25.31		34.04		8.74	0.11		
	SYBR	25.06		33.87		8.81	0.11		
ZW10 24	SYBR	25.73	0.42	31.39	0.53	5.66	0.94	0.81	0.11
		25.30		31.19		5.89	0.80		
	SYBR	26.14		32.19		6.06	0.71		
ZW10 48	SYBR	27.24	0.18	30.45	0.30	3.21	5.09	4.89	0.41
		27.07		30.27		3.19	5.16		
	SYBR	27.43		30.85		3.42	4.41		
ZW10 72	SYBR	27.42	0.25	31.15	0.27	3.73	3.57	3.50	0.08
		27.36		31.11		3.75	3.50		
	SYBR	27.82		31.61		3.79	3.41		

OsPR5									
Wells	Reporter	ACTIN	SD Avg Ct	PR5	SD Avg Ct	Δ Ct	Rel Exp	Avg Rel Exp	SD RX
CK 0	SYBR	28.27	0.43	31.83	0.36	3.56	TRUE	1.00	0.00
		27.95		31.17		3.22	1.00		

	SYBR	27.42		31.76		4.34	1.00		
CK 24	SYBR	29.26	0.12	31.29	0.23	2.02	11.62	12.87	1.09
		29.15		30.94		1.80	13.58		
	SYBR	29.02		30.84		1.82	13.43		
CK 48	SYBR	31.32	0.19	32.94	0.35	1.62	15.38	13.00	2.18
		31.40		33.49		2.09	11.09		
	SYBR	31.68		33.59		1.92	12.52		
CK 72	SYBR	28.89	0.81	30.78	0.48	1.89	12.71	11.71	2.54
		27.48		29.89		2.42	8.83		
	SYBR	28.85		30.65		1.80	13.61		
ZW10 0	SYBR	25.73	0.34	29.02	0.16	3.30	4.80	4.23	0.54
\		25.31		28.80		3.50	4.18		
	SYBR	25.06		28.73		3.67	3.72		
ZW10 24	SYBR	25.73	0.42	25.41	0.30	-0.33	59.19	53.24	7.18
		25.30		25.36		0.06	45.26		
	SYBR	26.14		25.91		-0.23	55.25		
ZW10 48	SYBR	27.24	0.18	27.99	0.30	0.75	28.05	29.18	2.64
		27.07		27.63		0.55	32.19		
	SYBR	27.43		28.22		0.79	27.30		
ZW10 72	SYBR	27.42	0.25	31.46	0.31	4.04	2.88	2.77	0.13
		27.36		31.44		4.07	2.80		
	SYBR	27.82		31.99		4.17	2.62		

OsWARK45									
Wells	Reporter	ACTIN	SD Avg Ct	WRKY	SD Avg Ct	Δ Ct	Rel Exp	Avg Rel Exp	SD RX
CK 0	SYBR	28.27	0.43	33.80	0.45	5.53	TRUE	1.00	0.00
		27.95		32.96		5.01	1.00		
	SYBR	27.42		33.11		5.69	1.00		
CK 24	SYBR	29.26	0.12	33.11	0.54	3.85	3.28	3.89	1.27
		29.15		33.10		3.96	3.04		
	SYBR	29.02		32.17		3.14	5.35		
CK 48	SYBR	31.32	0.19	31.46	0.39	0.14	42.82	38.69	5.23
		31.40		31.62		0.22	40.44		
	SYBR	31.68		32.20		0.53	32.80		
CK 72	SYBR	28.89	0.81	29.30	0.68	0.42	35.39	36.56	4.81
		27.48		28.02		0.54	32.44		

	SYBR	28.85		29.03		*0.17*	*41.85*		
ZW10 0	*SYBR*	25.73	*0.34*	31.57	*0.65*	*5.84*	*0.82*	*1.08*	*0.23*
\		25.31		30.58		*5.27*	*1.22*		
	SYBR	25.06		30.35		*5.29*	*1.20*		
ZW10 24	*SYBR*	25.73	*0.42*	28.48	*0.43*	*2.74*	*7.05*	*8.42*	*1.23*
		25.30		27.73		*2.42*	*8.80*		
	SYBR	26.14		28.46		*2.33*	*9.42*		
ZW10 48	*SYBR*	27.24	*0.18*	27.10	*0.16*	*-0.14*	*51.95*	*48.62*	*3.25*
		27.07		27.13		*0.06*	*45.46*		
	SYBR	27.43		27.39		*-0.04*	*48.46*		
ZW10 72	*SYBR*	27.42	*0.25*	33.01	*0.13*	*5.59*	*0.98*	*1.07*	*0.11*
		27.36		32.87		*5.51*	*1.04*		
	SYBR	27.82		33.13		*5.31*	*1.19*		
