# Supplementary material for: The antagonistic mechanism of Bacillus velezensis ZW10 against rice blast disease: Evaluation of ZW10 as a potential biopesticide
Source: PLoS One. 2021 Aug 27;16(8):e0256807. doi: 10.1371/journal.pone.0256807 (PMC8396770; doi:10.1371/journal.pone.0256807)
Supplement: S3 File — Row date for Fig 6. (PDF) [file pone.0256807.s004.pdf]

Leaf blast							
Water		Landy medium		Carbendazim		1% CFB	
leaves	disease index (%)	leaves	disease index (%)	leaves	disease index (%)	leaves	disease index (%)
6	82.000	6	84.000	7	13.714	3	14.667
7	78.857	6	84.000	5	16.800	5	16.000
6	76.000	7	78.286	9	20.889	6	19.333
4	88.000	5	75.200	4	18.000	3	17.333
5	76.800	5	78.400	3	20.000	6	17.333
8	86.000	8	91.500	8	20.000	8	19.000
10	66.800	10	78.800	7	17.143	3	17.333
3	96.000	4	82.000	4	21.000	6	16.667
6	76.667	5	79.200	4	17.000	4	17.000
6	72.000	4	84.000	8	20.000	3	20.000
6	86.000	6	80.000	9	19.111	6	18.667
6	75.333	8	89.500	8	19.500	3	17.333
4	94.000	4	94.000	4	22.000	4	18.000
5	79.200	8	79.500	5	18.400	5	16.800
6	82.000	6	68.667	4	18.000	4	21.000
2	92.000	9	80.889	6	16.000	3	18.667
6	84.667	6	75.333	4	21.000	4	17.000
2	84.000	5	78.400	2	16.000	5	16.800
7	72.571	6	83.333	5	16.000	5	16.800
10	81.200	10	68.800	3	18.667	10	19.200
4	82.000	4	90.000	4	19.000	9	15.556
8	86.500	8	82.000	7	16.000	8	17.500
4	88.000	6	81.333	8	18.500	3	17.333
6	78.667	6	71.333	3	17.333	3	21.333
6	78.000	6	79.333	6	14.667	6	17.333
7	84.000	7	77.143	7	17.143	7	17.714
7	73.714	6	83.333	5	19.200	5	16.800
8	84.500	8	91.000	8	20.000	8	18.500
6	77.333	6	81.333	2	10.000	4	17.000
6	86.667	7	77.714	3	21.333	5	19.200
3	77.333	5	83.200	7	17.714	3	16.000
8	87.500	8	84.500	8	19.500	8	18.000
7	68.000	6	76.667	5	18.400	5	19.200
9	83.111	7	76.000	7	18.857	7	16.571
3	96.000	6	77.333	4	18.000	3	17.333
5	78.400	3	80.000	7	15.429	5	16.800
6	80.667	7	77.714	7	17.714	3	18.667
4	84.000	10	74.400	8	18.500	4	19.000

2	76.000	6	81.333	3	17.333	10	16.400
6	78.000	6	78.667	9	20.000	6	16.667
6	73.333	3	85.333	7	19.429	4	18.000
10	78.400	8	84.500	4	18.000	10	13.600
7	86.286	7	77.714	6	15.333	4	14.000
5	81.600	6	85.333	3	22.667	5	15.200
3	85.333	9	77.333	5	19.200	4	17.000
9	83.111	7	78.857	7	20.000	7	20.000
7	73.714	6	77.333	5	16.000	5	17.600
6	73.333	4	82.000	6	20.667	4	16.000
10	72.800	10	68.800	10	14.400	10	18.800
9	79.111	9	85.778	7	20.000	7	18.286
8	96.000	9	84.000	8	18.500	8	16.500
8	84.000	8	86.000	8	18.500	8	19.500
5	84.000	7	80.000	6	17.333	4	15.000
6	82.667	4	88.000	6	18.000	3	17.333
4	73.000	6	86.000	5	13.600	2	18.000
5	81.600	5	77.600	6	16.000	4	18.000
9	84.000	7	80.571	7	16.000	7	18.286
6	72.000	6	80.000	7	13.714	4	21.000
10	67.200	10	73.600	4	21.000	4	17.000
6	72.667	7	73.143	9	20.889	6	17.333
3	77.333	6	73.333	3	21.333	3	18.667
7	76.571	7	83.429	7	20.000	7	19.429
6	68.667	6	78.000	6	15.333	6	18.000
5	82.400	5	77.600	6	16.667	4	15.000
5	73.600	4	84.000	3	17.333	9	14.667
5	88.000	7	85.714	5	19.200	5	16.800
5	89.600	3	77.333	6	19.333	6	19.333
3	85.333	5	76.800	5	18.400	6	16.667
8	82.500	8	81.500	8	18.000	7	17.714
7	83.429	7	72.000	7	14.286	4	12.000
5	78.400	5	72.000	4	19.000	4	19.000
7	77.714	6	86.000	5	14.400	5	18.400
7	84.571	5	78.400	6	16.667	5	20.800
7	77.143	6	86.667	5	15.200	5	15.200
6	76.000	5	75.200	7	16.000	3	17.333
10	67.200	10	70.000	4	19.000	10	18.800
6	78.000	5	84.800	4	18.000	4	17.000
7	72.571	6	76.667	5	16.800	5	19.200
8	91.000	6	78.667	7	17.714	7	21.714
7	78.286	5	73.600	7	16.571	7	18.857

4	80.000	6	68.000	4	18.000	5	19.200
3	80.000	5	76.000	6	16.667	3	17.333
5	83.200	10	79.200	6	16.000	4	16.000
10	68.400	5	83.200	10	20.000	10	20.000
4	86.000	7	75.429	6	18.667	5	20.000
6	73.333	10	65.600	9	21.778	6	14.000
10	84.800	8	81.000	4	18.000	10	14.000
8	75.000	6	85.333	6	18.000	5	17.600
7	79.429	7	73.714	5	17.600	5	16.000
6	75.333	8	87.500	9	22.667	6	16.000
8	82.000	10	67.200	8	18.000	7	17.714
10	73.200	5	75.200	10	19.200	10	18.400
5	81.600	7	76.571	6	16.000	4	15.000
6	80.667	7	78.857	9	21.778	6	18.000
7	80.571	5	75.200	7	19.429	7	20.571
3	85.333	7	79.429	2	18.000	5	17.600
6	72.667	8	86.500	7	13.143	3	18.667
9	87.556	5	69.600	7	17.714	7	17.143
6	75.333	8	84.000	4	19.000	4	18.000
8	84.500	7	80.571	8	16.500	7	16.571
6	85.333	8	86.000	4	21.000	6	21.333
5	87.200	10	72.000	5	19.200	5	20.000
10	82.000	10	67.200	10	15.200	10	14.000
10	80.800	7	74.857	10	18.000	10	20.000
6	72.667	10	66.800	9	22.222	6	16.667
10	79.200	5	83.200	4	8.000	10	21.600
5	75.200	8	84.000	6	17.333	6	17.333
8	84.500	10	85.600	8	17.500	8	20.000
10	75.200	9	85.333	10	10.000	11	22.545
4	82.000	7	77.143	6	16.667	2	16.000
9	85.778	5	81.600	7	18.286	7	20.571
4	76.000	5	82.400	5	16.800	12	16.333
5	78.400	7	78.286	4	17.000	4	19.000
9	75.111	5	74.400	7	14.857	7	20.000
6	80.667	6	80.667	3	18.667	6	19.333
6	80.667	5	73.600	9	20.000	6	18.667
3	82.667	4	76.000	6	18.000	4	17.000
5	79.200	6	82.667	5	18.400	3	21.333
5	84.800	9	85.333	6	18.000	4	17.000
8	81.500	5	84.800	8	19.000	8	18.500
4	86.000	6	72.667	4	14.000	5	17.600
7	79.429	10	72.400	5	16.800	5	16.800

10	78.400	10	71.600	4	15.000	4	20.000
5	86.400	8	81.000	4	17.000	4	18.000
8	79.000	6	78.667	7	18.857	7	17.143
4	74.000	8	84.000	6	18.667	5	15.200
8	86.500	6	70.000	8	20.500	8	17.500
7	78.857	8	75.000	5	15.200	5	16.800
8	89.000	3	74.667	8	16.500	8	19.000
6	80.667	10	67.200	6	18.000	6	16.667
10	74.800	6	80.667	5	16.800	10	12.800
7	75.429	5	87.200	5	18.400	5	18.400
7	81.143	8	84.000	7	16.571	5	18.400
3	77.333	10	66.800	5	16.800	4	18.000
10	60.800	6	84.000	10	13.600	10	18.000
5	84.800	6	78.667	7	15.429	4	18.000
2	94.000	7	79.429	5	18.400	6	16.667
9	85.778	8	88.000	7	15.429	7	19.429
9	83.556	6	78.667	7	14.286	7	17.714
6	80.000	9	75.556	3	17.333	4	21.000
2	88.000	9	83.111	3	14.667	5	17.600
8	78.500	6	81.333	8	18.000	8	18.000
5	86.400	8	89.500	5	19.200	3	16.000
9	84.000	5	84.800	7	19.429	7	18.857
3	82.667	10	66.400	4	19.000	3	18.667
10	83.600	5	80.800	6	16.667	10	13.200
5	83.200	7	73.714	6	16.667	4	14.000
6	74.667	6	82.000	9	20.889	6	18.000
6	78.667	5	76.000	9	19.556	6	14.000
5	77.600	6	78.667	4	15.000	4	18.000
6	71.333			6	14.000	6	16.667
4	73.000			5	19.200	6	17.333
3	80.000			3	22.667	5	20.000
				8	18.500	12	16.333
				6	18.667	5	17.600
				5	13.600	5	19.200
				3	20.000		
				6	18.667		
				5	16.800		
				4	18.000		
				7	17.714		

Disease index					
Water			Landy medium		
Mean	SD	N	Mean	SD	N
80.30259363	6.090	153	79.188	5.799863851	150.000
Carbendazim			1% CFB		
Mean	SD	N	Mean	SD	N
17.72080252	2.379	161	17.69521405	1.885156169	156

Panicle blast

Water		Landy medium		Carbendazim		1% CFB	
disease index / %	thousand seed weight	disease index / %	thousand seed weight	disease index / %	thousand seed weight	disease index / %	thousand seed weight
72.00	24.800	72.00	14.375	32.00	30.800	32.00	34.200
70.00	15.833	70.00	19.500	35.00	28.667	36.67	27.333
66.67	21.667	68.33	13.429	20.00	39.500	31.67	36.000
71.25	15.750	75.00	18.714	35.00	31.125	32.50	30.750
71.43	16.857	63.33	15.857	31.25	35.000	32.50	30.625
70.00	22.857	72.86	12.875	30.00	36.857	30.00	36.000
68.57	22.286	67.14	13.556	32.86	33.143	32.86	29.286
72.86	15.714	62.86	17.000	32.86	33.714	30.00	36.429
76.25	13.500	71.25	10.000	31.25	36.125	35.00	32.375
78.75	16.125	83.33	17.300	32.22	25.000	32.00	31.600
71.43	16.571	71.43	17.000	32.86	30.000	30.00	34.286
72.50	15.000	77.14	11.750	35.00	32.000	31.25	35.250
68.33	15.833	76.00	14.714	36.67	33.167	30.00	34.286
67.50	18.500	67.50	16.667	37.50	29.875	37.50	32.750
70.00	14.889	83.75	19.400	31.82	24.909	31.43	36.286
65.00	18.500	71.43	15.571	35.56	33.000	35.71	31.429
66.67	15.833	68.33	16.857	28.33	36.167	31.67	33.833
70.00	14.750	62.00	18.286	32.50	42.000	32.00	31.200
70.00	16.714	68.57	15.571	34.29	33.429	35.71	30.286
70.00	17.429	74.29	15.375	30.00	34.286	34.29	31.143
70.00	16.714	71.43	15.778	32.86	32.000	30.00	34.714
70.00		51.43	16.000	31.25		36.25	31.000
68.89		66.25	16.667	36.00		28.00	
70.00		73.33		40.00		32.22	

71.00	74.00	21.67	35.00
68.33	73.33	31.67	43.33
65.00	70.00	30.00	31.67
65.56	82.50	33.75	36.25
70.00	80.00	42.50	30.00
68.89	64.44	38.89	33.33
68.57	78.57	31.43	32.86
63.33	75.00	35.00	35.00
70.00	70.00	33.75	31.43
70.00	70.00	33.33	28.33
70.00	68.57	31.43	35.71
78.18	38.57	33.00	32.50
67.78	40.00	40.00	35.00
70.00	67.14	31.43	35.71
65.00	66.67	26.67	31.67
71.25	75.00	33.75	33.75
68.57	71.43	36.25	35.00
70.00	74.29	32.86	34.29
70.00	57.14	35.71	34.29
66.67	73.33	33.33	46.67
63.75	72.50	35.00	36.25
78.89	72.22	33.75	31.00
70.00	71.43	31.43	32.86
73.75	69.00	31.67	34.44
65.00	68.33	33.33	30.00
71.00	65.71	33.00	34.29
65.56	70.00	36.67	28.89
71.25	50.00	36.25	32.86
68.33	70.00	23.33	36.67
63.33	77.50	33.33	33.75
67.78	63.33	35.56	29.00
68.57	72.50	36.67	33.33
70.00	76.25	30.00	34.44
68.33	76.67	31.67	31.67
68.89	81.67	30.00	34.00
76.25	70.00	30.00	33.75
68.33	72.50	37.00	26.25
65.56	63.33	33.33	25.56
65.71	63.33	34.29	35.71
77.50	73.75	37.50	31.25
76.25	67.50	31.67	22.22
65.71	68.75	30.00	30.00

70.00	67.50	37.14	32.50
65.56	75.56	35.56	31.11
65.56	52.22	40.00	30.00
72.22	85.00	33.75	33.75
70.00	85.00	32.86	33.33
67.78	57.78	34.44	24.44
67.50	31.67	36.67	32.50
65.56	60.00	28.57	33.75
66.67	76.25	33.33	31.67
70.00	67.14	34.29	31.43
77.78	80.00	36.67	33.33
75.00	66.67	32.50	30.00
72.50	57.50	33.75	30.00
80.00	75.00	37.50	35.00
76.25	58.57	35.56	32.86
70.00	68.89	30.00	34.29
67.14	68.33	26.67	35.00
68.33	68.33	26.67	35.00
61.25	81.25	28.75	31.25
63.75	65.71	33.00	30.00
66.67	68.57	38.33	28.33
65.00	70.00	35.00	31.67
65.00	70.00	30.00	35.00
68.57	74.29	34.29	32.86
63.33	30.00	28.00	34.44
63.33	73.33	30.00	31.67
68.00	66.25	36.67	32.22
65.56	64.44	31.11	34.44
62.00	75.00	34.44	35.00
67.00	50.00	38.33	32.00
68.89	78.89	35.56	33.33
70.00	84.00	34.29	32.22
64.44	47.78	34.44	33.33
71.43	67.14	41.43	32.86
72.86	65.71	34.29	30.00
66.67	57.78	37.78	23.00
63.75	71.67	34.44	31.82
71.43	68.57	34.29	30.00
66.25	76.67	32.86	33.33
74.00	76.00	28.00	34.00
68.33	71.67	26.67	35.00
66.67	73.33	33.33	33.33

70.00	70.00	32.00	30.00
67.14	68.57	31.43	30.00
68.75	73.33	36.25	36.25
74.29	64.29	31.43	30.00
68.57	75.71	32.86	34.29
75.71	68.57	33.33	25.45
71.11	78.89	32.22	31.11
66.25	76.25	36.25	35.00
67.78	47.78	33.75	30.00
68.75	88.75	32.86	34.29
66.25	61.43	37.78	30.00
74.00	71.67	34.00	36.00
67.78	83.33	32.22	33.33
72.50	66.00	28.33	28.00
70.00	64.29	32.86	35.71
65.56	74.00	32.86	32.00
65.00	70.00	35.00	30.00
67.78	73.75	31.25	37.50
68.33	68.00	33.33	35.00
70.00	65.71	32.86	28.57
68.57	65.71	35.71	30.00
65.00	73.33	36.67	36.67
63.33	77.50	30.00	31.25
62.50	66.67	38.75	36.67
70.00	70.00	35.00	36.25
72.86	71.43	35.71	32.86
70.00	67.50	34.29	30.00
76.00	78.18	32.00	35.00
70.00	75.00	32.86	27.14
68.33	70.00	33.33	45.00
68.33	60.00	35.00	31.25
65.56	67.78	35.56	31.11
73.75	71.25	32.50	32.50
70.00	73.33	41.11	36.67
66.25	66.67	30.00	30.00
70.00	60.00	37.14	32.86
62.00	61.67	30.00	31.82
65.56	66.67	32.22	30.00
71.43	71.43	35.71	32.86
72.00	63.33	32.86	33.33
65.56	73.33		32.22
68.33			28.57

31.11
30.00

Disease index					
Water			Landy medium		
Mean	SD	N	Mean	SD	N
69.05925445	3.699282509	150	69.032407	9.197294374	149
Carbendazim			1% CFB		
Mean	SD	N	Mean	SD	N
33.37433456	3.446011295	148	32.56518664	3.288527538	152
Thousand seed weight / g					
Water			Landy medium		
Mean	SD	N	Mean	SD	N
17.43444822	2.925941895	20	15.74963768	2.301331159	23
Carbendazim			1% CFB		
Mean	SD	N	Mean	SD	N
32.89346011	4.032248283	21	32.7754329	2.45349832	22
